# Sodium-glucose cotransporter-2-induced euglycemic diabetic ketoacidosis unmasks latent autoimmune diabetes in a patient misdiagnosed with type 2 diabetes mellitus: a case report

**DOI:** 10.1186/s13256-020-02607-2

**Published:** 2021-02-14

**Authors:** Brian Vadasz, Mattan Arazi, Yousef Shukha, Ofir Koren, Riad Taher

**Affiliations:** 1 The Ruth and Bruce Rappaport Faculty of Medicine, Technion-Israel Institute of Technology, Haifa, Israel; 2 Internal Medicine Department E, Rambam HealthCare Campus, Ein IbrahimUmm alfahm, POD 4147, 300100 Haifa, Israel; 3 Department of Endocrinology, Rambam HealthCare Campus, Haifa, Israel; 4 Heart Institute, Emek Medical Center, Afula, Israel

**Keywords:** Diabetic ketoacidosis, Sodium-glucose cotransporter 2 inhibitors (SGLT2i), Latent autoimmune diabetes in adults (LADA)

## Abstract

**Background:**

Euglycemic diabetic ketoacidosis is an uncommon but life-threatening complication associated with the use of sodium-glucose cotransporter 2 inhibitors that causes lower than expected blood glucose levels typically seen in diabetic ketoacidosis.

**Case presentation:**

We present a case of 64-year-old Caucasian male patient previously diagnosed with type 2 diabetes treated with a sodium-glucose cotransporter 2 inhibitor who developed severe ketoacidosis. Serum glucose levels on initial presentation were slightly above normal baseline level. The patient was revealed to have latent autoimmune diabetes in adults.

**Conclusion:**

This case highlights the importance of prescribing sodium-glucose cotransporter 2 inhibitors to the correct patient population and the significance of accurately differentiating between various types of diabetes.

## Background

Sodium-glucose cotransporter 2 (SGLT2) inhibitors are a class of antiglycemic drugs that lower blood glucose concentration levels by preventing the reabsorption of glucose in the proximal tubule of the kidney, leading to glycosuria [1]. Examples of SGLT2 inhibitors are canagliflozin, dapagliflozin, and empagliflozin. They can be used as monotherapy or in combination with other antiglycemic agents [2].

One of the main side effects of these pharmacological agents is an increased risk of developing both genital infections and urinary tract infections [3]. SGLT2 inhibitors are often preferred among primary care providers because they improve glycemic control without significant risk of hypoglycemia and are associated with weight loss. Specifically, empagliflozin has been shown to reduce cardiovascular and all-cause mortality in patients with type 2 diabetes and established cardiovascular disease [4].

In addition, studies with empagliflozin, canagliflozin, and dapagliflozin have suggested that SGLT2 inhibitors reduce heart failure hospitalization and decrease the rate of progression of diabetic kidney disease [5, 6]. However, accompanied with their use is risk of diabetic ketoacidosis (DKA), a rare but serious adverse effect [7].

In May 2015 the US Food and Drug Administration (FDA) issued a warning indicating that treatment with SGLT2 inhibitors may increase the risk of euglycemic diabetic ketoacidosis (euDKA), while the American Association of Clinical Endocrinologists (AACE) and American College of Endocrinology (ACE) released a position statement in 2016 discussing a similar association [2, 8]. The FDA went further in only approving SGLT2 inhibitors for use in individuals with type 2 diabetes. In an 8-week study that included type 1 diabetes patients only, approximately 5% of patients treated with empagliflozin were withdrawn from the study because they developed DKA [9]. This association has also been reported in patients with type 1 diabetes who are being treated off-label with SGLT2 inhibitors [10]. This association is well documented, although the mechanism remains to be elucidated [11].

We present a case report of a 64-year-old male reported to have type 2 diabetes who developed euDKA while taking SGLT2 inhibitors, and was revealed to have latent autoimmune diabetes in adults (LADA).

## Case presentation

A 64-year-old Caucasian male with a 30-year duration of type 2 diabetes presented to the emergency department with generalized weakness, diffuse abdominal pain, nausea and vomiting, watery diarrhea, and reduced fluid intake for the past 3 days. His past medical history includes hypertension treated with amlodipine, untreated hyperlipidemia, and a subarachnoid hemorrhage in 2010. He is also treated with colchicine for unspecified joint pain.

Contrary to current guidelines, the patient’s diabetes was managed exclusively with short-acting insulin which was administered as needed without basal insulin. Six months before his admission, his HbA1c was measured at 9.3%. Per his primary care physician, 12.5 mg of empagliflozin, an SGLT2 Inhibitor, and 850 mg metformin hydrochloride were added as a combined treatment regimen to improve glycemic control. After introducing these agents, his current insulin dosage was reduced by 30%, and he continued to take short-acting insulin as needed up to his admission.

Upon admission, the patient denied fever, shivering, cough or shortness of breath, chest pain or palpitations, signs of urinary tract symptoms, and reported no signs of neurological impairment. On physical examination, the patient appeared weak and answered slowly. Pulse was 106 beats per minute (bpm), blood pressure 148/83 mmHg, oxygen saturation 100% on room air, deep and labored respiratory rate of 18 breaths/minute, temperature 36.6 °C. The calculated body mass index was 24.8 kg/m^2^. Abdominal examination revealed slight tenderness upon palpation of the epigastrium.

Laboratory studies upon arrival, summarized in Table 1, revealed an anion gap (25.3 mEq/L) metabolic acidosis (pH 7.04, bicarbonate 5.7 mEq/L,* p*CO_2_ 21 mmHg) with hyperglycemia (182 mg/dL) and ketonuria (150 mmol/L). Electrocardiogram displayed sinus tachycardia at 105 beats per minute with no signs of ischemia or arrhythmia. Chest X-ray was unrevealing.

**Table 1:** Laboratory results upon admission and follow-up

	Upon admission	Intensive care unit	Upon discharge	
Serum glucose (mg/dL) 70–99	182 mg/dL	166	234	
Blood urea nitrogen (mg/dL) 8.4–25.7)	10.3	4.1	11.6	
Creatinine (mg/dL) 0.7–1.3	1.40	0.88	0.62	
Sodium (mEq/L) 133–145	142	142	138	
Potassium (mEq/L) 3.5–5.3	4.7	4.1	3.8	
Chloride (mEq/L) 95–110	111	117	101	
Arterial blood gas				
Bicarbonate (mEq/L) 22–26	5.7	14.40	26	
Lactate (mmol/L) 0.0–1.3	1.2	0.60	N/A	
pH (7.35–7.42)	7.04	7.32	7.48	
*p*CO_2_ (mmHg) Anion gap (mEq/L) 3–11 35–45	21.0	28.0	35	
Base excess (mEq/L) − 2–2	− 23.30	− 17.30	2.8	
Anion gap (mEq/L) 3–11	25.3	10.6	11	
WBC count (neutrophil differential)	11.34 × 10^3^/μL, 91.8%	10.4 × 10^3^/μL	5.51 × 10^3^/μL, 64.4%	
Urinalysis				
Glucose (mg/dL)	> 1000	N/A	N/A	
Ketones (mmol/L)	150 mmol/L	N/A	N/A	
Leukocytes, nitrite	Negative	N/A	N/A	

*WBC* white blood cells,* PCO2* partial pressure of carbon dioxide

The patient's blood glucose was atypical for DKA, which prompted the emergency department to investigate other causes for the patient’s acute presentation. Urinary tract infection was ruled out by the lack of urinary complaints as well as a negative urinalysis for leukocytes and nitrites (Table 1). However, once DKA was suspected, the patient was started on standard solution of 5% dextrose in 0.45% normal saline, intravenous regular insulin and potassium. The SGL2 inhibitor and metformin were stopped. After initial treatment, the high anion gap metabolic acidosis did not improve substantially (anion gap 22 mEq/L, pH 7.07, *p*CO_2_ 15 mmHg, bicarbonate 4.6 mEq/L), and the patient was transferred to the intensive care unit. Lactate levels were normal, and osmolar gap was calculated at 10. In the intensive care unit, the patient was stabilized over 2 days with intravenous fluids, insulin, and potassium. Upon transferring from the intensive care unit, the anion gap was closed, and the patient was moved to the internal medicine department, where his insulin regimen was switched to subcutaneous basal bolus insulin (blood gases after transferring: bicarbonate 26 mEq/L, *p*CO_2_ 35 mmHg, pH 7.48, base excess 2.8 mEq/L, anion gap 7 mEq/L (Table 1).

Follow-up anti-glutamic acid decarboxylase antibodies were measured at 66 U/mL (normal < 5 U/mL), while anti-insulin antibodies and islet cell antibodies were negative. The patient was diagnosed with LADA. The patient was discharged in a stable state, and was recommended to follow up with a diabetes specialist and to undergo further ambulatory testing, including a follow-up HbA1c.

## Discussion

DKA is a metabolic complication of diabetes that can cause serious morbidity and mortality [11]. It is characterized by hyperglycemia, ketosis and acidosis, and occurs in states of absolute or relative insulin deficiency [11]. When deprived of glucose, the brain can metabolize either ketone bodies or amino acids as a source of energy [11]. DKA is precipitated by metabolically stressful events, such as prolonged fasting, illnesses, extensive exercise, and surgery, which can shift carbohydrate catabolism to fatty acid oxidation [8]. Ketogenesis occurs in the liver as a result of elevated insulin counter-regulatory hormones, leading to elevated ketone bodies and subsequent acidosis [11].

In rare instances, diabetic ketoacidosis can present with “lower-than-anticipated glucose levels” typically present in DKA, termed euglycemic DKA (euDKA) [2, 8]. EuDKA has been associated with the use of SGLT2 inhibitors, and is potentially dangerous as the acidosis may go undetected [12]. Patients with euDKA may present with increased plasma ketones and anion gap acidosis; however, their plasma glucose is typically < 14 mmol/L [13].

SGLT2 inhibitors lower blood glucose levels by inhibiting the reabsorption of glucose at the proximal tubule in the kidney, thereby inducing glycosuria. The half-life of SGLT2 inhibitors is between 11 and 13 hours, and their effect can persist for some time after discontinuation [14, 15]. Therefore, patients with diabetes on SGLT2 inhibitors require lower doses of insulin, which may further promote ketogenesis [11, 16]. While euDKA may be precipitated by factors that normally trigger DKA in diabetics, a systematic review by Burke *et al.* indicated that 9 out of 34 patients diagnosed with SGLT2 inhibitor-induced DKA did not have a precipitating factor and were later shown to have LADA [17]. After analyzing the FDA Adverse Event Reporting System (FAERS) for incidences of acidosis in patients treated with SGLT2 inhibitors, Blau *et al.* found a sevenfold increase in developing acidosis in patients with type 2 diabetes on the drug. About 70% of these patients had euDKA [18].

The true mechanism of SGLT2 inhibitor-induced ketoacidosis is not fully understood [11]. However, as patients with diabetes already have an elevated baseline of glucagon, adding SGLT2 inhibitors may further elevate glucagon levels in the blood [11]. Ferrannini *et al.* demonstrated that patients on 4-week treatment of an SGLT2 inhibitor had an increased glucagon to insulin ratio, in addition to reduced glucose oxidation and increased lipid oxidation [19]. Bonner *et al.* demonstrated that SGLT2 inhibitors caused glucagon secretion and gluconeogenesis in healthy mice [20]. In addition, they showed that SGLT2 expression levels were lower while glucagon levels were higher in T2DM compared to non-diabetics [20]. Finucane suggested that some individuals may have an uncommon variant of SGLT2 within their beta cells, which may be involved with GLUT2-mediated glucose transport. These transporters have a greater than normal affinity for SGLT2 inhibitors, thereby impairing glucose sensing and causing the loss of insulin secretion and lowering the threshold for euDKA [21].

Our patient’s acute presentation was misleading as he was not only labeled as a type 2 diabetic but his blood glucose was less than usually present in DKA. In addition, our patient was not obese, nor had characteristics of metabolic syndrome typically seen in individuals with type 2 diabetes; a family history of diabetes was unknown. Furthermore, our patient was undertreated with short-acting insulin only. The lack of continuous insulin availability may have contributed to ketogenesis and the development of DKA after initiation of the SGLT2 inhibitor.

DKA is uncommon in T2DM and our patient’s metabolic profile was atypical for insulin-resistant diabetes. Therefore, ancillary tests were ordered for our patient and revealed that he had an autoimmune diabetes. LADA is a progressive autoimmune insulitis characterized by the presence of autoantibodies, most commonly glutamic acid decarboxylase 65 (GAD65) autoantibodies, and occurs much later in life than type 1 diabetes [22–24]. Often misdiagnosed with T2DM, patients with LADA tend to be younger, have better metabolic profiles, lower BMI, worse HbA1c level, and require insulin more frequently than individuals with type 2 diabetes—characteristics seen in our patient [22]. In addition, patients with LADA often have other autoimmune diseases, such as autoimmune thyroiditis, compared to T2DM [22, 25].

A review of the literature demonstrated another patient who previously was thought to have T2DM but was revealed by have autoimmune diabetes after experiencing an episode of DKA on an SGLT2 inhibitor [14]. While ketoacidosis is more likely to occur in a low insulin state, the authors stress the importance of distinguishing T2DM from autoimmune diabetes, which presents with insulin deficiency, before starting a patient on an SGL2 inhibitor, in order to prevent the development of euDKA [14]. Another study completed by Erondu* et al*. showed that the incidence of DKA among patients with T2DM on canaglifozin was 0.07%. After the incident, half of the patients who suffered DKA and related adverse events tested positive for GAD65 autoantibodies or were diagnosed with LADA [26].

This case highlights the fact that patients with T2DM starting SGLT2 inhibitor treatment on top of insulin therapy should follow up regularly with their physician. LADA should be included in a differential diagnosis for this patient population, even before a presentation of DKA occurs.

## Conclusion and recommendations

This case demonstrates the necessity to accurately differentiate between the various types of diabetes, and to revisit a case when the current treatment protocol is ineffective. In addition, it highlights the potential for SGLT2 inhibitors to cause DKA, even in states of non-pathological glucose levels. While SGLT2 inhibitors are effective antiglycemic agents in type 2 diabetes, their use in type 1 diabetes has not been approved and may in fact be harmful [2]. Therefore, this case stresses the importance of prescribing SGLT2 inhibitors to the right population in addition to close monitoring and regular follow-ups for any adverse events. Simple screening tools to differentiate the phenotypically similar diseases LADA and T2DM are warranted for clinical use [14, 18].

Importantly, SGLT2-induced DKA, specifically euDKA, is not always associated with the typical signs and clinical manifestations such as dehydration induced by severe hyperglycemia. Therefore DKA in this patient population can be easily missed [25]. Understanding precipitating factors may also help primary care providers better identify patients at risk of developing SGLT2 inhibitor-related DKA. In an event of DKA in patients taking SGLT2 inhibitors, early diagnosis is imperative regardless of blood glucose levels [27].

Proper patient education is essential not only in identifying when an event of DKA is beginning but also in scheduling regular follow-ups with their primary care providers. The current guidelines recommend patients actively measure blood and urine ketones during acute illnesses, even with normal blood glucose levels [8, 12]. If symptoms of ketoacidosis occur, the FDA recommends that patients should discontinue their SGLT2 inhibitor and seek medical assistance immediately [2]. When managing patients with DKA on SGLT2 inhibitors, the AACE and ACE recommend stopping the drug, and continuing with normal DKA treatment [8].

## Data Availability

All data will be available upon acceptance.
